# A theoretical timeline for myocardial infarction: immunohistochemical evaluation and western blot quantification for Interleukin-15 and Monocyte chemotactic protein-1 as very early markers

**DOI:** 10.1186/1479-5876-12-188

**Published:** 2014-07-02

**Authors:** Emanuela Turillazzi, Marco Di Paolo, Margherita Neri, Irene Riezzo, Vittorio Fineschi

**Affiliations:** 1 Department of Forensic Pathology, University of Foggia, Ospedale Colonnello D’Avanzo, Viale degli Aviatori, n. 1, 71100 Foggia, Italy; 2 Department of Forensic Pathology, University of Pisa, via Roma 55, 56100 Pisa, Italy; 3 Department of Forensic Pathology, University “La Sapienza” of Rome, Viale Regina Elena 336, 00161 Roma, Italy

**Keywords:** Cytokines, Immunohistochemistry, Myocardial infarction, Timing, Western blotting

## Abstract

**Background:**

Experimental and human studies have demonstrated that innate immune mechanisms and consequent inflammatory reaction play a critical role in cardiac response to ischemic injury. Thus, the detection of immuno-inflammatory and cellular phenomena accompanying cardiac alterations during the early inflammatory phase of myocardial infarction (MI) may be an excellent diagnostic tool. Current knowledge of the chronology of the responses of myocardial tissue following the occurrence of ischemic insult, as well as the existence of numerous studies aiming to identify reliable markers in dating MI, induced us to investigate the myocardial specimens of MI fatal cases in order to better define the age of MI.

**Methods:**

We performed an immunohistochemical study and a Western blot analysis to evaluate detectable morphological changes in myocardial specimens of fatal MI cases and to quantify the effects of cardiac expression of inflammatory mediators (CD15, IL-1β, IL-6, TNF-α, IL-15, IL-8, MCP-1, ICAM-1, CD18, tryptase) and structural and functional cardiac proteins.

**Results:**

We observed a biphasic course of MCP-1: it was strongly expressed in the very early phase (0-4 hrs), to diminish in the early period (after 6-8 hrs). Again, our choice of IL-15 is explained by the synergism with neutrophilic granulocytes (CD15) and our study shows the potential for striking cytokine synergy in promoting fast, local neutrophil response in damaged tissues. A progressively stronger immunoreaction for the CD15 antibody was visible in the areas where the margination of circulating inflammatory cells was detectable, up to very strong expression in the oldest ones (>12 hours). Further, the induction of CD15, IL-15, MCP-1 expression levels was quantified by Western blot analysis. The results were as follows: IL-15/β-actin 0.80, CD15/β-actin 0.30, and MCP-1/β-actin 0.60, matching perfectly with the results of immunohistochemistry. Control hearts from traumatic death cases did not show any immunoreactivity to the pro-inflammatory markers, neither were there any reactions in Western blot analysis.

**Conclusions:**

Essential markers (i.e. IL-15, MCP-1) are suitable indicators of myocardial response to ischemic insult involving very early phase reaction (inflammatory response and cytokine release). In the very near future, proteomics may help clinicians and pathologists to better understand mechanisms relating to cardiac repair and remodeling and provide targets for future therapies.

## Background

From a clinical point of view, the term myocardial infarction (MI) can be used when there is evidence of myocardial necrosis in a clinical setting consistent with acute myocardial ischemia. Clinical features include electrocardiographic findings and elevated values of biochemical markers of myocardial necrosis [1]. From a pathological point of view, MI consists in a particular myocardial cell death due to ischemia. After the onset of myocardial ischemia, cell death is not immediate, but takes a finite period of time to develop. Complete myocytes necrosis is followed by a process leading to healed infarction. The symptoms and signs of MI may be confusing, and only rarely can this assessment be solved on the basis of clinical data [1].

**Figure 1:**
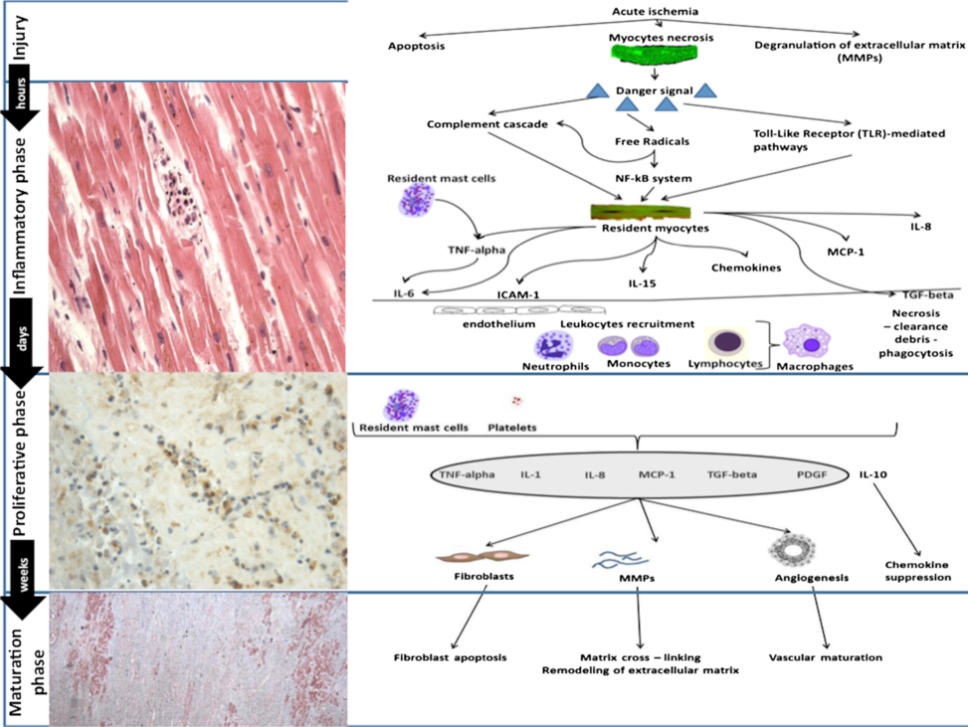
The time course of the inflammation, repair, and remodeling of the infarcted heart.

Experimental and human studies have demonstrated that innate immune mechanisms and consequent inflammatory reaction play a critical role in cardiac response to ischemic injury (Figure 1) [2-6]. Thus, the detection of immuno-inflammatory and cellular phenomena accompanying the cardiac alterations during early inflammatory phase of MI may be an excellent diagnostic tool. Before the influx of inflammatory cells becomes histologically detectable, the presence and nature of cellular and humoral mediators can be evaluated by immunohistochemistry. Attention should be focused on the immunohistochemical detection of different markers of myocardial response to ischemic insult. Humoral and cellular mediators have proved a worthwhile target for the postmortem diagnosis and timing of ischemia-induced cardiac injury [7-15]. Current knowledge of the chronology of the responses of myocardial tissue following the occurrence of ischemic insult, as well as the existence of numerous studies aiming to identify reliable markers in dating MI, induced us to investigate the myocardial specimens of MI fatal cases in order to better define the age of MI [16-18].

We performed an immunohistochemical study and a Western blot analysis to evaluate detectable morphological changes in myocardial specimens of fatal MI cases and to quantify the effects of cardiac expression of inflammatory mediators (CD15, IL-1β, IL-6, TNF-α, IL-15, IL-8, MCP-1, ICAM-1, CD18, tryptase) and structural and functional cardiac proteins (troponin I and troponin C).

## Methods

### Clinical data and tissue specimens

The clinical data and autopsy records of the 1260 autopsies performed at the Departments of Forensic Pathology of the University of Foggia and the University of Pisa (Italy) over the period 2001–2013 were evaluated, and 26 cases in which MI was indicated as cause of death were selected. We selected only cases with a well-defined clinical course (clinical symptoms, ECG and laboratory data), and in which postmortem examination confirmed the diagnosis of MI. The patients had a survival time ranging from 4–6 hours to more than 12 hours from the abrupt onset of typical symptoms. Twenty-five cases of instant death or with survival limited to 12 hours due to injuries from firearms without pathological cardiac involvement, were selected as the control group. In each case, the tissue samples obtained from the heart (standard seven specimens and additional samples taken from areas with macroscopic alterations, stained by hematoxylin–eosin and trichromic stains) were re-examined histologically. In addition, an immunohistochemical investigation of all the samples was performed utilizing a panel of antibodies (CD15, DAKO, Copenhagen, Denmark), IL-1β (Santa Cruz, CA, USA), IL-6 (Santa Cruz, CA, USA), TNF-α (Santa Cruz, CA, USA), IL-15 (R&D Systems, Minneapolis, MN, USA), IL-8 (Abcam, Cambridge, UK), MCP-1 (Santa Cruz, CA, USA), ICAM-1 (Santa Cruz, CA, USA), CD18 (Abcam, Cambridge, UK), tryptase (Novus Biologicals, Littleton, CO, USA). To obtain a better definition of early infarction, we matched the samples with very early markers of necrosis such as cellular antigen troponin C (Novocastra Leica Biosystems GmbH, Nussloch, Germany) and Troponin I (Thermo Fisher Scientific, Fremont, CA, USA). We used 4 mm-thick paraffin sections mounted on slides covered with 3-amminopropyl-triethoxysilane (Fluka, Buchs, Switzerland). Pre-treatment was necessary to facilitate antigen retrieval and to increase membrane permeability to antibodies anti-CD 15, IL-1β, MCP-1, IL-15, ICAM-1, CD 18, IL-8, troponin C and troponin I boiling in 0.25 M EDTA buffer, to antibodies anti-TNF-α boiling in 0.1 M Citric Acid buffer, to antibody anti- IL-6 and tryptase for 15 min. in Proteolytic Enzyme (Dako, Copenhagen, Denmark), at 20°C.

The primary antibody was applied in a 1:50 ratio CD 15, in a 1:4000 ratio IL-1β, in a 1:2000 ratio IL-6, in a 1:600 ratio TNF-α, in a 1:100 ratio IL-15, in a 1:500 ratio IL-8, ICAM-1 and MCP-1, in a 1:1000 ratio tryptase, in a 1:200 ratio CD 18, in a 1:3000 ratio troponin I and in a 1:6000 ratio troponin C and incubated for 120 min at 20°C. The detection system utilized was the LSAB + kit (Dako, Copenhagen, Denmark), a refined avidin–biotin technique in which a biotinylated secondary antibody reacts with several peroxidase conjugated streptavidin molecules. The positive reaction was visualized by 3.3-diaminobenzidine (DAB) peroxidation, according to standard methods. The sections were counterstained with Mayer’s haematoxylin, dehydrated, coverslipped and observed in a Leica DM4000B optical microscope (Leica, Cambridge, UK). A semi-quantitative evaluation of the immunohistochemical findings was made by two different investigators without prior knowledge. The reactions were graded as follows: 1. (0): not expressed, 2. (+): isolated and disseminated expression, 3. (++): expression in widespread foci, 4. (+++): widespread expression. All measurements were carried out using the same magnification of image (10×) and by the same two examiners. A third blind microscopic evaluator was involved to weigh the histological evidence. The samples were also examined under a confocal microscope, and a three-dimensional reconstruction was performed (True Confocal Scanner, Leica Biosystems GmbH TCS SPE).

### Western blot analysis

Western blot analysis was performed. Approximately 100 mg of frozen cardiac tissue was dissected and immediately transferred to the RIPA buffer with a protease inhibitor cocktail, and homogenized on ice utilizing the homogenizer Silent Crusher. The homogenate was centrifuged (12000 RPM for 10 min at 4°C). The supernatant was collected, estimated by Qubit Fluorometer (Invitrogen), and boiled for 5 min, at 95°C. Cardiac total protein extracts (approximately 40 μg/lane) were run on 4-15% SDS PAGE at 80 V for about 2.5 hr. For Western blot, proteins from SDS (sodium dodecyl sulfate) gels were electrophoretically transferred to nitrocellulose membranes in mini trans blot apparatus (1 hr at 250 mA). Non-specific binding was blocked by incubating membranes in Western blocker solution for 1 hr at room temperature. The membranes were incubated with primary antibodies anti-TNF- α, IL-1β, IL-6, IL-8, MCP-1, CD 18, IL-15, CD 15, Tryptase, ICAM-1diluted in Western blocker solution, in 1:400 ratio for TNF-α, in 1:200 ratio for IL-1β, IL-15, CD 15, IL-6, IL-8, MCP-1, CD 18, Tryptase, ICAM-1 overnight at 4°C. Blots were washed with PBS (Phosphate Buffered Saline/Tween-20) and then incubated for 1 hr at room temperature with HRP (horseradish peroxidase)-conjugated secondary antibodies diluted in Western blocker solution, in a 1:2000 ratio. Membranes were washed with PBS/Tween-20, and the immune reaction was developed in IMMUNOSTAR Kit Western C (Bio-Rad laboratories) and then visualized by Chemiluminescent detection methods. The light was then detected by photographic film. The image was analyzed by Versadoc (Bio-Rad laboratories), which detected the chemiluminescent blots of protein staining.

### Statistical analysis

A semi-quantitative evaluation of the immunohistochemical findings and gradation of the immunohistochemical reaction were described with an ordinal scale and the median value reported. An analysis of variance for the non-parametric data between groups was performed using the Kruskal-Wallis test. When differences were found to be significant, an analysis between the unmatched groups was performed with a Dunn's Multiple Comparison post hoc test. Significance level was set to 5% (SPSS ver. 16.01 for Windows - SPSS Inc., Chicago USA).

## Results

**Figure 2:**
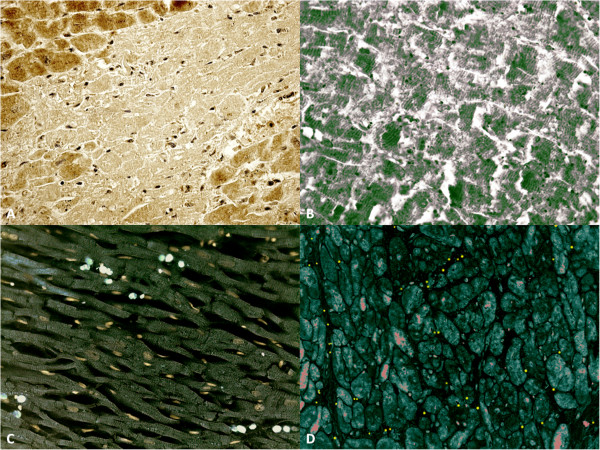
**Time course of cytokine and extracellular matrix protein up-regulation in myocardial infarcts at 4hs. (A)** Troponin I is markedly unexpressed in a large area of infarcted myocardium (Ab-anti TnI). **(B)** The confocal microscopic observation of early (4hs) myocardial infarction showed a large area of TnC depletion (in green the TnC reaction). **(C)** Double immunohistochemical reaction (IL-15 and CD15) evidenced an intense IL-15 (bleu) and sporadic CD15 (white) reactions in the infarcted zone. **(D)** Double immunohistochemical reaction (MCP-1 and CD15): MCP-1 reaction (red) is intense as well as CD15 (yellow) in the infarcted zone after 4 hrs.

Clinical data and histological results were compared to assemble the MI cases in chronological homogenous groups. The microscopic observation of myocardial samples showed the following chronological differences of the myocardium. In the group of very early MI (approximately 0–6 hours from the onset of ischemia related symptoms), mild myofiber eosinophilia and elongation of sarcomeres and nuclei were fairly evident (loss of contraction/atonic death of myofibers) as well as prominent contraction band necrosis. No histological signs of polimorphonuclear margination (PMN) were visible. In the same cases an immunohistochemical investigation revealed a mild positivity of CD15, tryptase, IL-1β, IL-6, TNF-α, IL-8, CD18, ICAM-1 and intense IL-15 and MCP-1 reactions in the infarcted zone matched by the immunodepletion of negative markers of necrosis (cellular antigen troponin) (Figure 2).

**Figure 3:**
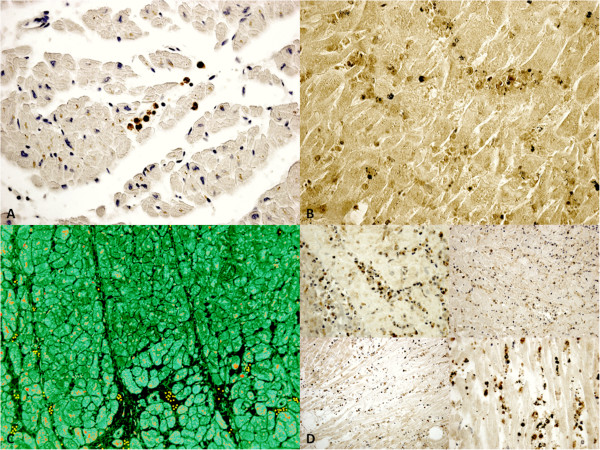
**Time course of cytokines and extracellular matrix protein up-regulation in myocardial infarcts at 6-12hs. (A)** Little foci of myocardial necrosis (<6 h) with intravasal and interstitial CD15 reaction (brown reaction); **(B)** Double immunohistochemical reaction (IL-15 and CD15): foci of myocardial necrosis (>6 h) with intensive expression of IL-15 and CD15. **(C)** Double immunohistochemical reaction (MCP-1 and CD15) showed a diffuse positivity (red and yellow respectively) after 6 hrs. **(D)** CD15 reaction after 6 hrs, after 8 hrs, after 10 hrs, and 12 hrs.

**Figure 4:**
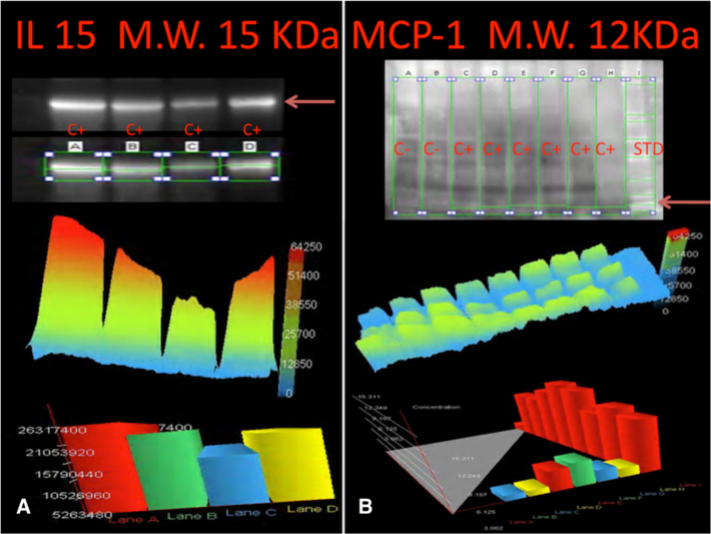
**Western blot analysis. (A)** Immunoblot analysis demonstrated the presence of IL-15, with a molecular weight of approximately 15 kDa, obtained from the cardiac tissue and the graphic overview of results based on the light intensity and the number of pixels detected, which shows a quantified intensity at 4 hours. **(B)** Immunoblot analysis demonstrated the presence of MCP-1, with a molecular weight of approximately 12 kDa, obtained from the cardiac tissue. A graphic overview of results based on the light intensity and the number of pixels detected, showing the concentration at 4 hours.

In the group of early infarction (approximately 6–8 hours from the onset of ischemic symptoms and signs) margination of PMN leukocytes that include neutrophils and monocytes was detectable in vessels at the periphery of the necrotic zone along with infiltration of these elements into the ischemic issue. A crowd of PMN was visible along a line between infiltrated and non-infiltrated necrotic myocardium in large areas of necrosis. Immunohistochemistry confirmed the nature of the inflammatory cells. A progressively stronger immunoreaction for the CD15 antibody was visible in the areas where the margination of circulating inflammatory cells was detectable up to very strong expression in the oldest ones (>12 hours) (Figure 3) (Table 1).Further, the induction of CD15, IL-15, MCP-1 expression levels was quantified by Western blot analysis. The results were as follows: IL-15/β-actin 0.80, CD15/β-actin 0.30, and MCP-1/β-actin 0.60, matching perfectly with the immunohistochemistry results (Figure 4). Control hearts from traumatic death cases did not show any immunoreactivity to the pro-inflammatory markers, neither were there any reactions in Western blot analysis.

**Table 1:** Semi-quantitative evaluation and statistical analysis of the immunohistochemical findings and gradation of the immunohistochemical reaction in the heart samples

**Antibody**	**Group A (≤4-6 hours from the onset of ischemia related symptoms)**	**Group B (≥6-8 hours from the onset of ischemic symptoms and signs)**	**Group C controls**	**Significant level**	**Significance levels**^**1**^
CD15	+	+++	-	A vs C	**
				B vs C	***
				A vs B	**
IL-1β	+	+++	−	A vs C	**
				B vs C	***
				A vs B	**
IL-6	+	+++	-	A vs C	**
				B vs C	***
				A vs B	**
TNF-α	+	+++	-	A vs C	**
				B vs C	***
				A vs B	**
IL-15	+++	+	−	A vs C	***
				B vs C	**
				A vs B	**
IL-8	++	+++	−	A vs C	**
				B vs C	***
				A vs B	*
MCP-1	+++	+	-	A vs C	***
				B vs C	**
				A vs B	**
ICAM-1	+	++	−	A vs C	*
				B vs C	**
				A vs B	*
CD18	-	-	−	A vs C	ns
				B vs C	ns
				A vs B	ns
Troponin C	+	+++	_	A vs C	**
				B vs C	***
				A vs B	**
Troponin I	+	+++	_	A vs C	**
				B vs C	***
				A vs B	**
Tryptase	+	+++	−	A vs C	**
				B vs C	***
				A vs B	**

^1^Intensity of immunopositivity was assessed semiquantitatively in the scale 0–4 as follows: 1. (0): not expressed, 2. (+): isolated and disseminated expression, 3. (++): expression in widespread foci, 4. (+++): widespread expression.

NS: *p* > 0.05; *: *p* < 0.05; **: *p* < 0.01; ***: *p* < 0.001.

These two chronological groups are homogenous and the described morphological changes were classified when clinical data coherently supported the microscopic chronological modification.

Our results are summarized in Table 2 with possible classification based on histological, immunohistochemical age determination and western blotting quantification of MI.

**Table 2:** Histological/immunohistochemical and Western blotting age determination of MI

		
Cell death	Up to 30 minutes – 1 hour	Cytoplasm and mitochondrial swelling and dissolution of the cristae mitochondriales (electron microscopy); loss of contraction with stretching of the myocardium in flaccid paralysis, resulting in a very early elongation of sarcomeres and nuclei; mild myofiber eosinophilia. Foci of contraction band necrosis. At immunohistochemistry loss of cellular antigen (myoglobin and cardiac troponin) is detectable earlier than the accumulation of plasma markers (C5b-9 complex, fibronectin).
Inflammatory phase	4-6 hours	Mild positivity of immunoreaction (tryptase, CD15, IL-1β, IL-6, IL-8, TNF-α) and stronger reactivity for IL-15, MCP-1 in areas where depletion of cellular antigens (myoglobin and cardiac troponin) is detectable within 30 – 40 minutes from ischemia.
	6-8 hours	Necrosis of the infarcted area becomes more evident; a crowd of polymorphonuclear leukocyte infiltration from the periphery is evident. General and intense eosinophilia of myofibers. Interstitial oedema. Immunopositivity to the antibodies anti tryptase, CD15, IL-1β, IL-6, IL-8, IL-15, TNF-α becomes stronger and ubiquitously widespread. MCP-1 decrease as intensity in respect to the first hours.
		Pronounced necrosis of the infarcted areas; strong evidence of PMN margination with further leukocyte penetration of the infarct area. Strong immunopositivity to anti CD15 antibodies was observed.

## Discussion

Our data show that some investigated parameters, such as CD15, IL-15, MCP-1, have a significantly different expression in the groups being studied (very early and older infarction). Monocyte chemotactic protein (MCP)-1, expressed by the main inflammatory and stromal cells, such as endothelial cells, mediates the recruitment of mononuclear cells, modulates monocyte and lymphocyte phenotype and regulates fibrous tissue deposition and angiogenesis. Its expression is upregulated after proinflammatory stimuli and tissue injury [19]. We observed a biphasic course of MCP-1: it was strongly expressed in the very early phase (0-4 hrs), diminishing in the early period (after 6-8 hrs). Again, our choice of IL-15 is explained by the synergism with neutrophilic granulocytes (CD15) and our study shows the potential for striking cytokine synergy in promoting fast, local neutrophil response in damaged tissues. Neutrophils are major players in inflammation and are known to express all components of the IL-15.

It is well known that the response of the myocardium to ischemic insult can be divided into overlapping phases: the inflammatory phase, the proliferative phase and the maturation phase. Sudden induction of ischemia triggers a series of events that culminate in the death of ischemic cardiomyocytes; the necrotic muscle elicits an inflammatory cascade that serves to clear the infarct from dead cells and matrix debris, and ultimately results in the healing and replacement of the damaged tissue with scarring [3,20].

The pathological pictures of the later stages (i.e. proliferative and maturation) are quite well-known: starting from 2–3 weeks from MI, pronounced peripheral granulation tissue with sprouted capillary blood vessels, fibrocytes, fibroblasts, lymphocytes, some plasma cells, macrophages, possibly siderophages, and some granulocytes become increasingly apparent. From 5 weeks to 2–3 months, histological findings included collagen fiber or scar tissue with endothelially-coated blood vessels of varying density, siderophages still possible, loose infiltration with lymphocytes, very few plasma cells, and scant granulocytes [21-23]. In the inflammatory phase, immediately following myocardial ischemia, chemokines and cytokines are induced in the infarct and marked leukocyte infiltration is noted. Neutrophils and macrophages clear the wound from dead cells and matrix debris.

If, on one hand, the pathophysiological mechanisms underlying the very early phase of myocardial response to ischemia are well known [3,5], on the other hand, the weakest part of the available morphological evidence to date appears to be right in this phase when cellular reaction is not yet histologically detectable.

The finding of IL-15, MCP-1 expressions in the cardiac specimens of early infarction confirm a considerable amount of existing data that show the role of humoral (cytokines and inducible chemokines, complement, and toll-like receptors) and cellular (monocytes, macrophages, dendritic cells, T cells, mast cells, platelets, endothelial cells) mediators in the initial healing phases following cardiomyocytes death [4,6,17,24-31]. Activation of cytokine cascades in the infarcted myocardium was established in numerous studies. In experimental models of myocardial infarction, the induction and release of the pro-inflammatory cytokines are regularly described [16,17]. The multifunctional, overlapping and often contradictory effects of the cytokines have hindered understanding of their functional role in cardiac injury and repair. In the infarcted area, a marked cytokine up regulation is present due to various mechanisms like Reactive Oxygen Species (ROS) generation, complement activation, and NF-κB (nuclear factor kappa-light-chain-enhancer of activated B cells) activation potently stimulate cytokine mRNA (Messenger Ribonucleic acid) synthesis in both resident and blood-derived cells [16-18].

Many authors have attempted to assess the histological age of MI, classifying the chronological phenomena into phases using histochemical methods only. The first attempts were established by White et al. [32] who graded “the speed of healing” as follows: [1, represents the reaction and death of the heart muscle cells as the result of the blocking of the blood supply during the first 24 to 48 hours before the appearance of many invading cells; 2, the invasion by multitudes of white cells which come to clear away the debris of the very necrotic myocardium during the first week after the initial 24 to 48 hours; 3, the stage of rapid disappearance of the damaged muscle cells, replacement of polymorphonuclear cells by mononuclears, and new vascularization of the infarct in the second week; 4, the laying down of the scar with the beginning of the appearance of collagen in the third and fourth weeks; and 5, the completely healed scar with much collagen after 2 or 3 months]. An attempt to define early infarct necrosis was afterwards performed by Lodge-Patch [33] who focused on the character of the cellular exudate as the most valuable feature in estimating the age of a young infarct. The results constitute important findings concerning morphological changes and chronological organization of myocardial infarct. Nuclear changes in muscle fibers, necrosis and phagocytosis of muscle, interstitial edema, neutrophils and polimorphonuclears infiltration were found to be a typical pattern at a time of 6–8 hours from the onset of acute symptoms [33]. These findings suggested that the age of a cardiac infarct could be told from its histological appearances. However, myocardial infarcts could not be accurately dated before 6–8 hours from the onset.

More recently, the first histological change, visible within 10 minutes of onset, has been recognized to be the intense hypereosinophilia of the hypercontracted myocardial cells with rhexis of the myofibrillar apparatus into cross-fiber, anomalous, and irregular pathological bands, formed by segments of hypercontracted sarcomeres with scalloped sarcolemma [20,34]. The lesion is unrelated to ischemia. Its presence in acute coronary syndromes is probably due to catecholamines released within the myocardium as a reflex response [35] to regional asynergy of the infarcted or perinfarcted zone, a hypothesis that is supported by the abolishment of contraction bands and ventricular fibrillation with beta-blocking agents in experimental myocardial infarction and in reperfusion necrosis [36]. They may trigger catecholamine myotoxicity linked with ventricular fibrillation and acting through free radical mediated lipid peroxidation with intramyocellular Ca2+ influx. Contrary to the general opinion that excess catecholamines produce cardiotoxicity mainly through binding to adrenoceptors, there is increasing evidence that catecholamine-induced deleterious actions may also occur through oxidative mechanisms [37,38], which undoubtedly occur during myocardial reperfusion after ischemia [17]. Interstitial edema and wavy fibers have been also described [39] in very early infarct but these findings are very unspecific [21]. No pathognomonic histological sign is visible within 6 to 8 hours of survival after onset when PMN infiltration starts. Consequently, despite several attempts, no suitable histological criteria have materialized into forensic practice.

## Conclusions

We agree that “optimal assessment of cardiac remodeling in myocardial infarction requires a wide range of functional, molecular, histological, and proteomic investigations. Understanding the temporal window of each cellular and molecular response is critical for designing studies exploring the mechanisms of repair and remodeling of the infarcted heart” [2]. An approach based on the pathophysiochemistry of myocardial infarction may be useful to aid or support “traditional” pathomorphological observations [40]. Essential markers (i.e. IL-15, MCP-1) are suitable indicators of myocardial response to ischemic insult involving very early phase reaction (inflammatory response and cytokine release). The histological age determination of myocardial infarction is an important task of forensic medicine and requires thorough knowledge of the general and specific pathology of myocardial infarction. So, we need to investigate the pathology of myocardial infarction as a cause of sudden cardiovascular death. Chronological evaluation needs the analysis of parameters within a histological specimen such as any measurement of the number and size of cellular expressions/modifications. Sometimes, we need to judge the medical practice in the cases of in-hospital myocardial infarction among patients who were treated for other diseases and to determine the causal relationship with myocardial infarction as cause of death. In the very near future, proteomics may help clinicians and pathologists to better understand mechanisms related to cardiac repair and remodeling and provide targets for future therapies.

## Abbreviations

MI: Myocardial infarction; CD15: Cluster of differentiation 15 (neutrophils); IL-1β: Interleukin-1 beta; TNF-α: Tumor necrosis factor alpha; IL-15: Interleukin-15; IL-8: Interleukin-8; MCP-1: Monocyte chemotactic protein-1; ICAM-1: Intercellular adhesion molecule 1; CD 18: Cluster of differentiation 18 (integrin beta-2); ECG: Electrocardiography; DAB: Diaminobenzidine; RIPA: Radio-immunoprecipitation assay; PBS: Phosphate buffered saline; HRP: Horseradish peroxidase; PMN: Polymorphonuclear; ROS: Reactive oxygen species; NF-kB: Nuclear factor kappa-light-chain-enhancer of activated B cells; mRNA: Messenger ribonucleic acid.

## Competing interests

The authors declare that they have no competing interests.

## Authors’ contributions

ET and VF conceived of the study, participated in its design and coordination and helped to draft the manuscript. MN and IR carried out the histological and immunohistochemical studies and drafted the manuscript. MDP participated in the design of the study and performed the statistical analysis. All authors read and approved the final manuscript.
